# Association of Dietary Fatty Acids with Blood Lipids is Modified by Physical Activity in Adolescents: Results from the GINIplus and LISA Birth Cohort Studies

**DOI:** 10.3390/nu10101372

**Published:** 2018-09-25

**Authors:** Carla P. Harris, Andrea von Berg, Dietrich Berdel, Carl-Peter Bauer, Tamara Schikowski, Sibylle Koletzko, Joachim Heinrich, Holger Schulz, Marie Standl

**Affiliations:** 1 Institute of Epidemiology, Helmholtz Zentrum München–German Research Centre for Environmental Health, 85764 Neuherberg, Germany; carla.harris@helmholtz-muenchen.de (C.P.H.); heinrich@helmholtz-muenchen.de (J.H.); schulz@helmholtz-muenchen.de (H.S.); 2 Dr. von Hauner Children’s Hospital, University Hospital, LMU of Munich, 80337 Munich, Germany; sibylle.koletzko@med.uni-muenchen.de; 3 Department of Pediatrics, Research Institute, Marien-Hospital Wesel, 46483 Wesel, Germany; avb.rodehorst@gmx.de (A.v.B.); berdel.vonberg@t-online.de (D.B.); 4 Department of Pediatrics, Technical University of Munich, 80804 Munich, Germany; carl-peter.bauer@drv-bayernsued.de; 5 IUF—Leibniz Research Institute for Environmental Medicine, 40225 Düsseldorf, Germany; tamara.schikowski@IUF-Duesseldorf.de; 6 Institute and Outpatient Clinic for Occupational, Social and Environmental Medicine, Inner City Clinic, University Hospital of Munich (LMU), 80336 Munich, Germany; 7 Allergy and Lung Health Unit, Melbourne School of Population and Global Health, The University of Melbourne, Victoria 3010, Australia; 8 Comprehensive Pneumology Center Munich (CPC-M), German Center for Lung Research, 81337 Munich, Germany

**Keywords:** fatty acids, carbohydrates, blood lipids, physical activity, sedentary, lifestyle, MVPA, adolescents, substitution

## Abstract

The role of consuming different types of fatty acids (FA) at the expense of carbohydrates (CHO), on the blood lipid profile of adolescents is largely unknown, as is the modulating effect of different levels of physical activity (PA). Children from the GINIplus and LISA birth cohorts, with complete data on dietary FA (assessed by food-frequency questionnaires), objectively-measured PA (assessed by accelerometers) and blood lipids (lipoprotein cholesterol and triglycerides) at age 15 years, were included (*N* = 837). Sex-stratified associations between dietary FA and blood lipids were assessed by linear regression in substitution models which represented isocaloric replacements of CHO with saturated FA (SFA), monounsaturated FA (MUFA), n-3 polyunsaturated FA (PUFA) or n-6 PUFA. To assess the interactions with PA, analyses were then performed stratified by tertiles of different PA levels (sedentary, lifestyle, moderate-to-vigorous (MVPA)). Both sexes presented a significant inverse association between MUFA and triglycerides, and females a direct association between n-3 PUFA and high-density lipoprotein. Stratifying by PA tertiles, associations were mainly restricted to participants with the lowest levels of lifestyle PA, or the highest time spent sedentary. The effects of dietary FA on the lipid profile vary in an activity-specific manner, emphasizing possible synergistic roles of diet and PA.

## 1. Introduction

Disturbed blood lipid profiles, characterized by low levels of high-density lipoprotein (HDL) cholesterol, and high levels of low-density lipoprotein (LDL) cholesterol and triglycerides (TAG), can be observed as early as childhood and are believed to track into adulthood [1,2], representing an early risk for cardiovascular disease (CVD) [3]. Diet and physical activity (PA) are among the most important modifiable lifestyle factors known to influence blood lipid levels and have become central targets in the prevention of chronic diseases [4]. However, common correlations among different dietary components as well as the likely interplay between diet and PA (both behavioral and metabolic) [5], highlight the need to interpret their roles as a synergy of interactions rather than as individual exposures with independent health effects.

With respect to the role of diet in lipoprotein metabolism and CVD risk, fats and carbohydrates (CHO) are frequently discussed in adult populations [6,7,8]. In particular, dietary saturated fatty acids (SFA) have been suggested to negatively impact blood lipids by increasing LDL cholesterol particles [9,10]. There is convincing evidence in adults for an improved lipoprotein profile when replacing dietary SFA with polyunsaturated (PUFA) fatty acids and, to a lesser extent, with monounsaturated fatty acids (MUFA) [11]. On the other hand, substituting CHO for SFA has been shown to reduce HDL cholesterol levels and to increase TAG [12]. The focus of healthy eating has indeed undergone a notable shift towards reducing dietary CHO, especially refined starches and sugars [13,14,15]. Moreover, emerging analyses failing to observe a link between SFA and CVD have cast doubt on the widely-postulated detrimental role of dietary SFA [16,17]. Amidst often opposing stances among scientists, there is a danger for consumers to be misled, consequently replacing one possibly unhealthy dietary behavior for another [18]. Current dietary practices typically present an inverse correlation between fat and (simple) CHO intakes [19]. Hence, a reduction in energy intake from CHO most likely entails a simultaneous increase in fat. It is important to understand the implications of such an exchange during adolescence, a stage characterized by rapid growth and development [20] as well as drastic behavioral changes [21] which often track into adulthood.

We have previously observed that theoretically replacing dietary SFA with the same calories from CHO resulted in increased LDL cholesterol, TAG and total to HDL cholesterol ratio (total:HDL) levels in females [22]. This suggests that young, healthy populations may indeed benefit (in a sex-specific manner) from reducing CHO intakes, even when accompanied by an increase in SFA consumption. Nonetheless, considering the bulk of evidence, it is likely that other types of (unsaturated) fatty acids would lead to improved lipid profiles when consumed in place of CHO. In adults, replacing dietary CHO with MUFA or PUFA prompted the greatest health benefits in terms of changes in TAG levels and in total:HDL cholesterol [23,24]. So far, metabolic responses to such dietary modifications in adolescents have not been investigated. Additionally, processes following the ingestion of nutrients, such as digestion, absorption, uptake into tissues and metabolism, can play a pivotal role in the ensuing circulating fatty acids (including lipids integrated in cell membranes and present as free fatty acids in the circulation) [25], and likely affect the resulting blood lipoprotein profile. Given its ability to influence metabolic health [26], a modulatory role of habitual PA is plausible and should not be neglected. Indeed, HDL cholesterol levels have been found to increase significantly with PA, whereas TAG levels are lowered [27]. Even light PA has been observed to produce favorable metabolic responses [28,29], while sedentary behavior is associated with a range of detrimental health outcomes [30]. Studies assessing the effect of dietary fatty acids on blood lipids should therefore take into account possible interactions with different levels of PA.

To our knowledge, the integrated role of dietary fatty acid intake and PA with respect to blood lipids during adolescence has not been addressed. Therefore, the present study aimed to assess the association of different types of dietary fatty acids (when theoretically replacing dietary CHO) with blood lipids in a large population of 15-year-olds; as well as the possible modifying role of different levels and duration of objectively-measured PA.

## 2. Materials and Methods

### 2.1. Participants

**Figure 1:**
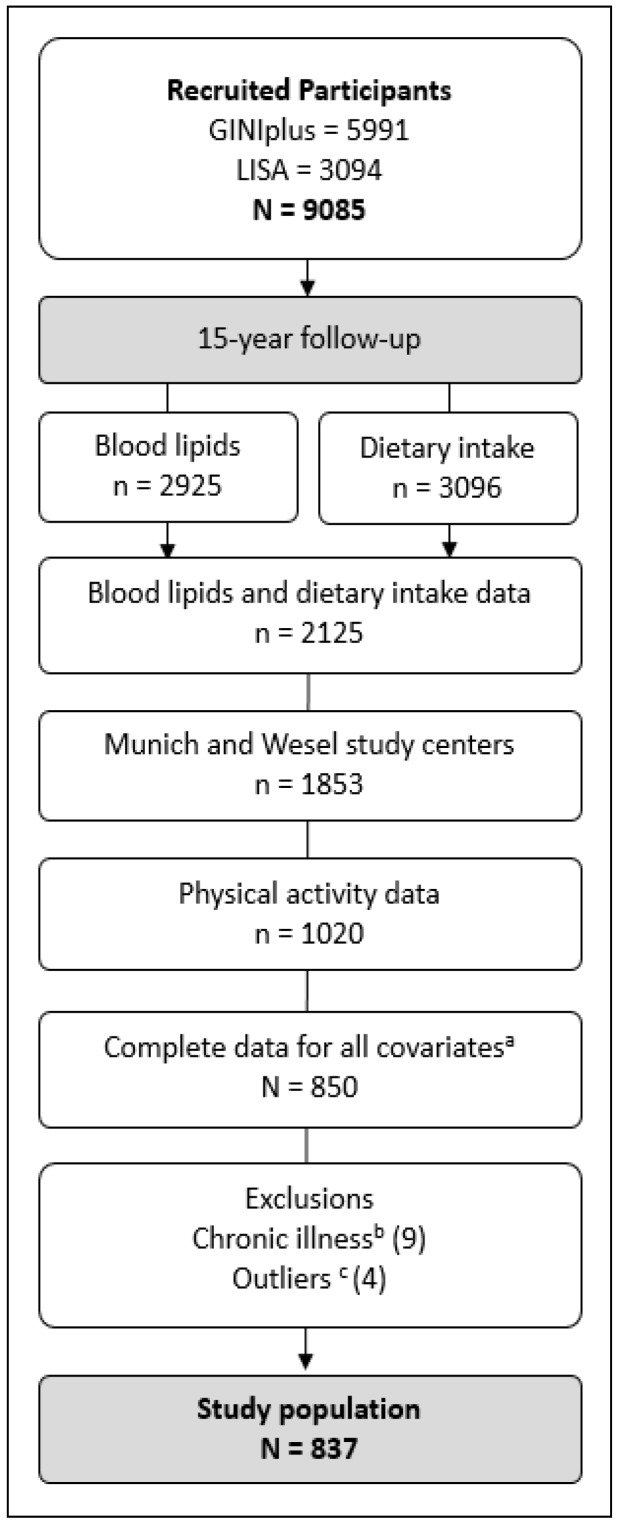
^a^ Covariates: Total energy intake, BMI, pubertal stage, fasting status, parental education, study (arm), region, age, sex (for stratified analyses). ^b^ Chronic illness: Diabetes (*N* = 3), celiac disease (*N* = 3), cancer (*N* = 2), Crohn’s disease (*N* = 1). ^c^ Outliers: Outliers in n-3 PUFA intake (*N* = 1), in n-6 PUFA intake (*N* = 2) and in MVPA (*N* = 2), visually identified using descriptive plots.

The present study was based on data obtained from 15-year-old adolescents participating in the GINIplus (German Infant Nutritional Intervention plus environmental and genetic influences on allergy development) and LISA (Influence of Life-style related factors on the development of the Immune System and Allergies in East and West Germany) German birth cohort studies (Figure 1). Details concerning recruitment and follow-up of participants have been described previously and can be found in earlier publications [31,32]. In brief, healthy, full-term newborns were recruited from selected obstetric clinics in Germany: In Munich and Wesel between 1995 and 1998 for the GINIplus study (*N* = 5991), and additionally in Leipzig and Bad Honnef between 1997 and 1999 for the LISA study (*N* = 3094—originally 3097 but three removed consent). Both cohorts are population-based, however, GINIplus consists of an observation arm and an intervention arm. For the intervention arm, newborns with a family history of allergy were invited and randomized to receive one of three hydrolyzed formulas or cow’s milk. The aim was to compare the effect of the different formulae on allergy development. Participants who declined to participate in the intervention trial were included in the observation arm, along with those with no family history of allergy. Information on selected exposures and health outcomes was collected by means of questionnaires and medical examinations, carried out at various follow-up assessments. The collection of data relevant to the present analyses is described in detail below. GINIplus and LISA were approved by their local ethics committees (Bavarian Board of Physicians, University of Leipzig, Board of Physicians of North-Rhine-Westphalia) and written consent was obtained from all participants’ families. Since PA was assessed in Munich and Wesel only, this analysis is restricted to subjects from Munich (*N* = 4413) and Wesel (*N* = 3390).

### 2.2. Blood Lipids

Blood samples were collected at the 15-year follow-up physical examination. The concentrations (mmol/L) of total cholesterol, LDL cholesterol, HDL cholesterol, and TAG were measured in serum using homogenous enzymatic colorimetric methods on a Modular Analytics System from Roche Diagnostics GmbH Mannheim according to the manufactures instructions (Roche Diagnostics GmbH Mannheim). External controls were used in accordance with the guidelines of the German Society of Clinical Chemistry and Laboratory Medicine. The ratio of total cholesterol to HDL cholesterol (total:HDL) was calculated by dividing total cholesterol by HDL cholesterol.

### 2.3. Dietary Intake

Dietary intake was assessed by means of a self-completed food frequency questionnaire (FFQ), designed to estimate food and nutrient intake over the past year, and specifically for the estimation of total energy, fatty acid and antioxidant intake in school-aged children. Briefly, the FFQ comprised a list of 80 food items, for which participants were asked to report average consumption frequency and portion sizes. A quality control procedure was applied based on recommendations by Willett, et al. for data cleaning in nutritional epidemiology [33], and is described in detail elsewhere [34]. Total daily energy intake (in kcal/day) and the intakes of SFA, MUFA, n-6 and n-3 PUFA, protein, carbohydrate and alcohol were calculated (in g/day) based on the German Food Code and Nutrient Database (BLS) version II.3.1 [35]. Macronutrient intakes were converted to kcal/day by applying the Atwater general factor system [36]. Each nutrient was expressed as its percentage contribution towards total daily energy intake (% EI), calculated as the ratio of energy from each nutrient to total daily energy intake, multiplied by 100.

### 2.4. Physical Activity

PA was measured at age 15 years using triaxial accelerometers (ActiGraph GT3X, Pensacola, FL, USA), worn on the dominant hip for seven consecutive days. Participants for accelerometry were recruited from the study centers Munich and Wesel. This included all of the GINIplus cohort and 64% of the LISA cohort (all from Munich and Wesel) taking part in the 15-year follow-up. The accelerometry protocol, data management and quality control have been described previously in detail [37,38]. Briefly, participants were asked to keep an activity diary to monitor time of getting up and going to bed, and to control for non-wear time as well as the plausibility of the recorded accelerometer data. After passing quality control, at least 10 h of recorded time (or 7 if subjects were awake for less than 10 h) were necessary for a recorded day to be considered valid. Subjects were required to have at least three valid recorded weekdays and one valid weekend day. Measured accelerations were converted into activity counts and stored at 1 Hz (resampled from 30 Hz). Activity counts were then classified into one of four intensity levels (sedentary, light, moderate, and vigorous PA) on a minute-by-minute basis, estimated according to the uniaxial cut-offs published by Freedson et al. [39]. For the current analyses, three levels of PA were evaluated: Sedentary, light (representing lifestyle PA), and MVPA (the sum of moderate and vigorous PA). Average minutes per day spent on the different PA levels were calculated for each individual by dividing total recorded minutes by the number of valid recorded days.

### 2.5. Statistical Analyses

Statistical analyses were carried out separately for females and males. Participants were excluded from the analyses if they had a chronic illness affecting diet or if they presented outliers in either an exposure or outcome variable, visually identified using descriptive plots. Subject characteristics at age 15 years were described by means (standard deviation) and medians (25th–75th percentile) for normally-distributed and skewed continuous variables, respectively, or counts (%) for categorical variables. Differences in characteristics between females and males were tested by Student’s t-test and Wilcoxon rank sum test, for continuous variables (normally-distributed and skewed, respectively), and by χ^2^-test for categorical variables.

Linear regression was used to assess the association of different types of dietary fatty acids with blood lipid parameters (LDL cholesterol, HDL cholesterol, TAG, total:HDL cholesterol). In order to assess associations as the theoretical replacement of dietary CHO with different fatty acids, a substitution model approach was applied, in which a model was fit including the different dietary fatty acids (SFA, MUFA, n-6 PUFA, n-3 PUFA), and adjusting for all other energy bearing nutrients (protein, alcohol) with the exception of CHO (the nutrient being replaced). In this way, the energy intakes of fats, protein, and alcohol are held constant; and by additionally including total energy intake in the model it is possible to interpret the resulting coefficients for each fatty acid as its theoretical substitution for an equal amount of energy (% EI) from CHO, being the only energy-bearing nutrient not accounted for in the model. Given the high inter-correlation typically present amongst dietary components [40], we calculated correlation coefficients between pairs of fatty acid variables, using Pearson’s product-moment correlation coefficient. A high positive correlation was observed between SFA and MUFA (r = 0.8 and r = 0.7, in females and males respectively). By linearly regressing SFA onto MUFA and vice-versa, we computed residuals (SFA_RESID_ and MUFA_RESID_) which were uncorrelated with each other [41]. To avoid multicollinearity, these residuals were included in the model as a stand-in for the original variable only when acting as a covariate (i.e., when assessing the effect of replacing CHO with n-6 PUFA or n-3 PUFA). When assessing the effect of replacing CHO with SFA, SFA was included in its original form as the main exposure variable, and MUFA_RESID_ was included in place of MUFA, along with all other covariates, and vice versa. All models were adjusted for potential confounders, including: Region (Munich; Wesel), study (LISA; GINI intervention; GINI observation), exact age at blood sampling (years), body mass index (BMI, in kg/m^2^ calculated from height and weight measurements obtained during physical examination, unless unavailable (*N* = 71), in which case obtained from the 15-year follow-up questionnaire), total daily caloric intake (kcal/day), parental education (based on highest level achieved; low: ≤10th grade; high: >10th grade), pubertal stage (based on self-rated pubertal development scale [42]: Early-; mid-; late-; post-pubertal), and fasted blood sampling (yes; no). Given the skewed distribution of LDL cholesterol, TAG and total:HDL cholesterol, these variables were log-transformed prior to analyses in order to obtain normal distributions. Effect estimates from regression analyses were then back-transformed from the log scale into means ratios (MR) and 95% confidence intervals (95% CI). This approach was performed in line with previous analyses [43,44]. The MR can be interpreted as the percentage change in the mean of the outcome variable (LDL cholesterol, TAG or total:HDL cholesterol) corresponding to a one unit increase in the exposure of interest (in this case, an interquartile range (IQR) increase in dietary SFA, MUFA, n-6 PUFA or n-3 PUFA when theoretically replacing CHO). For the assessment of HDL cholesterol, results are presented as regression coefficients (β) per IQR increase in the relevant exposure variable, along with their 95% confidence interval (95% CI). In a second step, the analyses were repeated with further adjustment for different levels of PA (sedentary, lifestyle PA and MVPA, in separate models). Finally, we tested for interactions and carried out the same analyses stratified by tertiles of the different physical activity levels. A two-sided α-level of 5% was considered significant; the stratified analyses were corrected for multiple testing using Bonferroni correction: The α-level was divided by 3 as the data were analyzed by tertiles (α-level = 5/3 = 1.7%). Within our sample we observed that TAG levels were significantly lower in fasting than in non-fasting subjects (median = 0.84 mmol/L vs. 1.08 mmol/L in females, and 0.86 mmol/L vs. 1.20 mmol/L in males), and hence performed additional sensitivity analyses to check for possible differences in results when stratifying by fasting and non-fasting subjects. All statistical analyses were conducted using R (www.r-project.org) (R Foundation for Statistical Computing, version 3.4.4, Vienna, Austria) [45].

## 3. Results

### 3.1. Study Population and Basic Characteristics

The present study analyses were based on data from 837 participants (472 females and 365 males). Figure 1 displays a summary of the derived study population. From those initially recruited (*N* = 9085), blood lipids, dietary intake and PA data were available from 1020 participants at the 15-year follow-up. Complete data for all adjustment variables were provided by 850 participants. Of these, nine participants were excluded due to chronic illness (3 due to diabetes, 3 due to coeliac disease, 2 due to cancer, and 1 due to Crohn’s disease). Finally, four participants were excluded from the analyses as they presented clear outliers, visually identified using descriptive plots (1 outlier in n-3 PUFA intake, 1 in n-6 PUFA intake and 2 in MVPA). Basic characteristics of the study population are described in Table 1, separately for females and males. Females had significantly higher LDL cholesterol and HDL cholesterol levels than males, and lower total:HDL cholesterol. Fatty acid intakes were similar between both sexes, with SFA ranging around 13% EI, MUFA around 11% EI, n-6 PUFA around 4% EI and n-3 PUFA around 0.6% EI. Males spent significantly more time in both lifestyle PA and MVPA than females, whereas females spent more time sedentary.

### 3.2. Analyses of Associations of Dietary Fatty Acids (When Theoretically Replacing CHO) with Blood Lipids 

Results of analyses assessing the effects on blood lipids of increasing levels of different dietary fatty acids at the cost of CHO are displayed in Table 2. When replacing CHO with MUFA, TAG levels were decreased in both sexes (females: MR = 0.94 (95% CI = 0.89; 1.00), *p*-value = 0.049; males: MR = 0.87 (95% CI = 0.82; 0.94), *p*-value < 0.001). Additionally, in females, n-3 PUFA was directly associated with HDL cholesterol levels (β = 0.07 (95% CI = 0.03; 0.12), *p*-value = 0.002). Further adjusting for different levels of PA did not alter the observed associations. However, when adjusting for MVPA in males, a significant direct association between MUFA and HDL cholesterol was observed (β = 0.06 (95% CI = 0.00; 0.11), *p*-value = 0.047), which in the main analyses was only borderline significant (*p*-value = 0.064). Exact values obtained with additional adjustment for different PA levels can be found in Supplementary Table S1.

### 3.3. Analyses Stratified by Tertiles of Different PA Levels (Sedentary, Lifestyle PA, MVPA)

**Figure 2:**
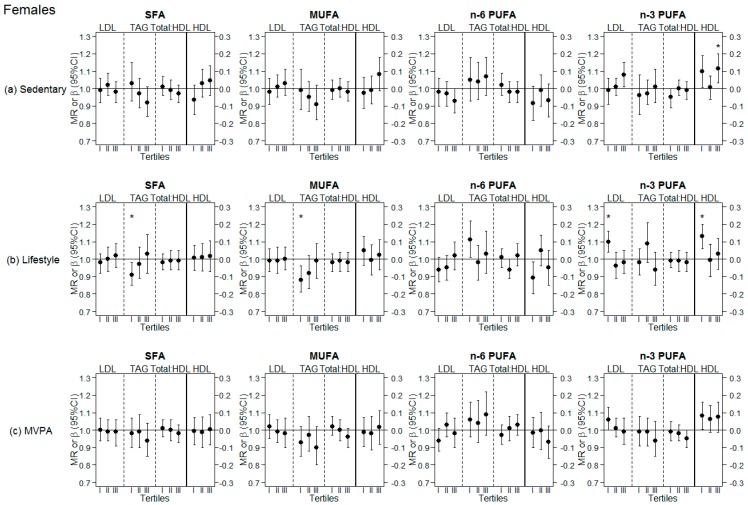
Analyses stratified by tertiles of different physical activity (PA) levels in females. (**a**) Sedentary: Top row, analyses stratified by tertiles of sedentary behavior; (**b**) Lifestyle: Middle row, analyses stratified by tertiles of lifestyle PA; (**c**) MVPA: Bottom row, analyses stratified by tertiles of moderate-to-vigorous PA (MVPA); LDL: Low-density lipoprotein cholesterol; HDL: High-density lipoprotein cholesterol; TAG: Triglycerides; Total:HDL: Total cholesterol to HDL ratio; SFA: Saturated fatty acids; MUFA: Monounsaturated fatty acids; PUFA: Polyunsaturated fatty acids; MR: Means ratio (for effects on LDL, TAG and Total:HDL, on left side of continuous black line); β: Beta coefficient (for effects on HDL, on right side of continuous black line); * significant associations following Bonferroni correction for multiple testing (*p*-value < 0.017). Tertiles (I, II, III): Tertiles of the respective physical activity level (Sedentary, Lifestyle PA, MVPA).

**Figure 3:**
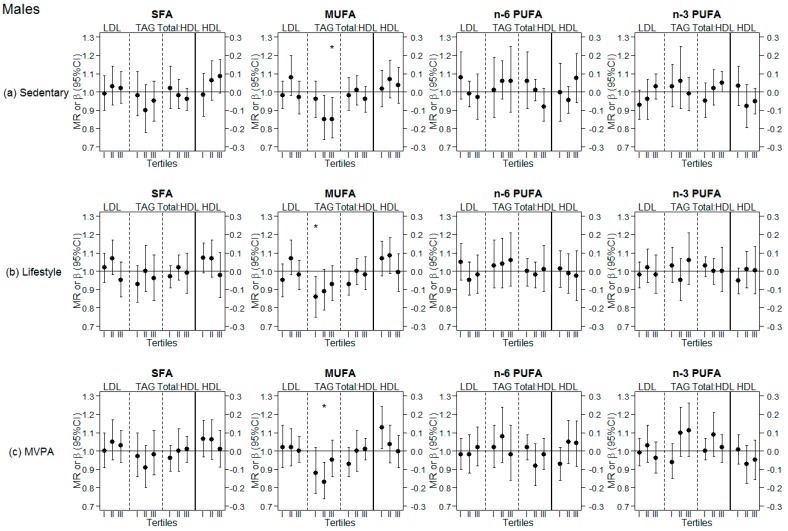
Analyses stratified by tertiles of different physical activity (PA) levels in males. (**a**) Sedentary: Top row, analyses stratified by tertiles of sedentary behavior; (**b**) Lifestyle: Middle row, analyses stratified by tertiles of lifestyle PA; (**c**) MVPA: Bottom row, analyses stratified by tertiles of moderate-to-vigorous PA (MVPA); LDL: Low-density lipoprotein cholesterol; HDL: High-density lipoprotein cholesterol; TAG: Triglycerides; Total:HDL: Total to HDL cholesterol ratio; SFA: Saturated fatty acids; MUFA: Monounsaturated fatty acids; PUFA: Polyunsaturated fatty acids; MR: Means ratio (for effects on LDL cholesterol, TAG and Total:HDL, on left side of continuous black line); β: Beta coefficient (for effects on HDL cholesterol, on right side of continuous black line); * significant associations following Bonferroni correction for multiple testing (*p*-value < 0.017). Tertiles (I, II, III): Tertiles of the respective physical activity level (Sedentary, Lifestyle PA, MVPA).

Results of the analyses stratified by tertiles of different PA levels are presented for females and males in Figure 2 and Figure 3, respectively. Exact values are displayed in Supplementary Tables S2 and S3. A significant interaction was observed with sedentary behavior when assessing the association between dietary SFA (at the expense of CHO) and HDL cholesterol in females (*p*-int = 0.045), and when assessing the association of MUFA with TAG in males (*p*-int = 0.045). Females in the highest tertile of sedentary time presented a significant positive association between n-3 PUFA and HDL cholesterol (β = 0.12 (95% CI = 0.03; 0.20), *p*-value = 0.007). Similarly, females in the lowest tertile of lifestyle PA presented a positive association between n-3 PUFA and HDL cholesterol (β = 0.13 (95% CI = 0.06; 0.20), *p*-value < 0.001), as well as with LDL cholesterol (MR = 1.10 (95% CI = 1.04; 1.16), *p*-value = 0.001). Furthermore, in this subgroup of low lifestyle PA, a significant inverse association was observed between SFA and TAG (MR = 0.91 (95% CI = 0.85; 0.98), *p*-value = 0.013) and between MUFA and TAG (MR = 0.88 (95% CI = 0.81; 0.96), *p*-value = 0.004). No association between fatty acids and blood lipids was observed in females when stratified by MVPA tertiles. In males, stratifying by tertiles of different levels of PA revealed a significant inverse association between MUFA and TAG only in the subgroups with either the highest sedentary time (MR = 0.85 (95% CI = 0.75; 0.97), *p*-value = 0.015) or the lowest lifestyle PA time (MR = 0.86 (95% CI = 0.75; 0.97), *p*-value = 0.015), as well as in the middle tertile of MVPA (MR = 0.83 (95% CI = 0.74; 0.94), *p*-value = 0.003).

### 3.4. Sensitivity Analyses Stratified by Fasting Status

In females (fasted *N* = 203, non-fasted *N* = 269), the effect size of the associations between MUFA and TAG remained almost unaltered regardless of fasting status, although associations were no longer statistically significant, possibly due to reduced power (fasted: MR = 0.93 (95% CI = 0.85; 1.01), *p*-value = 0.082; non-fasted: MR = 0.94 (95% CI = 0.87; 1.02), *p*-value = 0.132). Males (fasted *N* = 187, non-fasted *N* = 178) in the fasted group indicated a stronger inverse association between MUFA and TAG than previously observed (MR = 0.83 (95% CI = 0.76; 0.90), *p*-value < 0.001), whereas the non-fasted group presented no association (MR = 0.97 (95% CI = 0.88; 1.07), *p*-value = 0.568). Furthermore, an association was observed in fasted males between SFA and TAG (MR = 0.89 (95% CI = 0.81; 0.97), *p*-value = 0.008). When considering only fasted individuals, the inverse associations between SFA and TAG and between MUFA and TAG in the lowest tertile of lifestyle PA, were even stronger than previously observed in both sexes (SFA in females: MR = 0.88 (0.81, 0.96), *p*-value = 0.004; MUFA in females: MR = 0.82 (0.74, 0.91), *p*-value = 0.001; SFA in males: MR = 0.82 (0.72, 0.93), *p*-value = 0.003; MUFA in males: MR = 0.77 (0.68, 0.87), *p*-value < 0.001). The association between MUFA and TAG in males in the highest sedentary tertile was also stronger (MR = 0.78 (0.66, 0.93), *p*-value = 0.006), whereas no significant associations were present in any of the MVPA tertiles.

## 4. Discussion

The present cross-sectional study used 15-year follow-up data from two large German birth cohorts to investigate the role of consuming different types of dietary fatty acids at the expense of CHO, on the blood lipid profile of adolescents. Furthermore, possible modifications of associations by different levels of PA (sedentary behavior, lifestyle PA and MVPA) were evaluated using objectively-measured habitual PA data.

We have previously found that replacing dietary SFA with CHO results in a prospective increase in LDL cholesterol, TAG, and total:HDL cholesterol levels in female adolescents [22]. The opposite might be expected when CHO is replaced with SFA, however, this was not observed in the present analyses. Dietary recommendations for children and adolescents focus largely on limiting SFA intakes [11,46], yet we observed no detrimental changes in blood lipids with increasing dietary SFA at the expense of CHO. Nevertheless, possible adverse effects on other cardiometabolic risk factors such as low-grade inflammation [47], which was not assessed in the present study, must not be overlooked. On the other hand, in a large systematic review and regression analysis, Mensink reported reduced TAG and higher HDL cholesterol and LDL cholesterol levels, when CHO was replaced with SFA in adults [24]. Furthermore, replacing CHO with MUFA or PUFA resulted in higher levels of HDL cholesterol and reduced levels of LDL cholesterol, TAG and total:HDL cholesterol [24]. When CHO was isocalorically replaced with MUFA in our adolescent population, a reduction in TAG levels was observed in both females and males. This is in line with the above-mentioned findings, although differences in other blood lipid markers were not evident. The role of elevated TAG as an independent risk factor for cardiovascular disease is controversial [48], nevertheless there has been renewed interest in TAG as a marker of remnant cholesterol, which is considered a possible causal player in cardiovascular disease development [49,50]. The observed associations between MUFA intake and TAG could therefore prove relevant for the design of early preventive measures to be implemented in adolescents. With respect to dietary PUFA, we observed an increase in HDL cholesterol with n-3 PUFA in females, but no differences with n-6 PUFA. To our knowledge, the separate roles of n-6 and n-3 PUFA have not yet been addressed in the context of replacing dietary CHO. Most studies agree that replacing SFA with PUFA (n-6 and n-3), can lead to an improved blood lipid profile [11]. Furthermore, the replacement of CHO with any type of fatty acid has been reported to increase HDL cholesterol in adults, although in contrast to the present findings in females, effects diminish with increasing unsaturation [10].

Our stratified analyses indicated that PA modifies the association between dietary fatty acids and blood lipids in adolescents. In females, associations observed between MUFA and TAG were restricted to participants with the lowest levels of lifestyle PA. Females in this subgroup also presented an inverse association between SFA and TAG and positive associations of n-3 PUFA with LDL cholesterol and HDL cholesterol. Most of these associations are in line with existing data. A reduction in TAG levels following the replacement of CHO with SFA, is well documented [11]. Evidence for TAG-lowering effects of long-chain n-3 PUFA has been frequently reported [51], and although we did not observe such an effect in our study, levels of HDL cholesterol are known to be inversely correlated with TAG [50]. Furthermore, several studies have reported elevated LDL cholesterol levels with n-3 PUFA intake [52,53,54], as was observed in the present study. While differing levels of MVPA did not play a significant role in females, the strongest association between MUFA and TAG was seen in males grouped in the middle tertile of MVPA. Although significant inverse associations were also observed in males spending the longest time sedentary and in those with the lowest level of lifestyle PA, a greater effect size was seen for MVPA, than that observed for the other activity levels. A more potent effect of MVPA than light PA per unit time has been previously reported [55]. PA plays a role in the maintenance of insulin sensitivity and the partitioning of nutrient energy to metabolically active tissues [56]. The resulting alterations to blood lipids are believed to occur due to short-lived improvements in transport and disposition of dietary fat, occurring postprandially; therefore, regular, frequent activity has been recommended to benefit lipoprotein metabolism [29]. It is unclear why an association was observed for the middle MVPA tertile but not the highest tertile. Nevertheless, as a whole, the present findings suggest that adolescents (and particularly females) who are the least active in their daily lives are more susceptible to changes in diet involving the substitution of dietary fatty acids for CHO. Sedentary behavior has been observed to be adversely associated with cardiometabolic health, independent of MVPA [57,58,59]; and may at times also act as a better predictor of poor health than MVPA [60]. Light (lifestyle) activity has also been shown to have protective properties relevant to cardiovascular risk [61]. In the present study population, lifestyle PA covered a much larger proportion of waking hours than MVPA, which represented an average of 43 and 35 min per day, in males and females, respectively. Therefore, lifestyle PA is usually responsible for a substantial proportion of expended energy, and likely also reduces sedentary time. Sedentary behavior has been shown to cause a decrease in muscle lipoprotein lipase (LPL) activity in animal studies, which is reversed after reestablishing moderate ambulatory activity [62]. This effect of light PA is yet to be verified in humans. The biological mechanisms through which lifestyle PA can improve cardiometabolic health are, hence, not entirely understood. Nevertheless, given that LPL is needed for the lipolysis of TAG in circulating lipoproteins, and that dietary CHO elevates TAG levels [63], it seems plausible that adolescents with low lifestyle PA would be less capable of sustaining healthy lipoprotein metabolism (through reduced LPL activity), and would indeed benefit from dietary fatty acids replacing CHO (MUFA or n-3 PUFA). On the other hand, such dietary modifications may not be relevant in the more active individuals.

Differences between females and males in the association between dietary fatty acids and blood lipids have been observed previously in the GINIplus and LISA population [22]. The reasons for these differences could potentially be related to sex-specific dietary patterns, hormones or pubertal stage. A greater proportion of females in our study were in the late and post-pubertal stage whereas most males were still in mid-late puberty. It has been shown that physiological insulin resistance occurs during puberty [64], suggesting that females in the present study may have been more vulnerable to the potentially adverse effects of carbohydrates, and were therefore more prone to benefit from replacing dietary CHO with fatty acids. On the other hand, males were more physically active than females (lifestyle and MVPA), which may have placed them at a possible metabolic advantage. Nevertheless, we also considered the role of fasting and non-fasting blood TAG levels, as these were significantly lower in fasted than in non-fasted subjects. Observed associations with TAG tended to be stronger in fasted subjects; and, particularly in non-fasted males, associations were completely absent. These analyses indicated that fasting status plays a relevant role, and that non-fasted blood lipid measurements may partly obscure existing associations with TAG, especially in adolescent males. Given the lack of studies on the association of dietary fatty acids with blood lipids in adolescent populations, the comparison of our results is mostly limited to findings in adults. Based on our findings, adolescents seem less prone to dietary-induced changes in blood lipids, and associations observed in adults are hence not transferable to adolescents. Further studies in younger populations are warranted to confirm this. Discrepancies may also be due to age-related differences in dietary behaviors such as the relative intakes of different macronutrients or the types of foods typically consumed. PA is also likely to play a relevant role, as was reflected by the present analyses. Our analyses addressing the modification of associations with PA highlight the complexity of the body as a biological system, where the effects of diet are likely to be highly dependent on the metabolic status of the individual.

### Strengths and Limitations

The present study benefited from the thorough assessment of various crucial behavioral and health-related parameters, including habitual dietary intake, physical activity, and blood lipids, in a large sample of 15-year-old females and males. Our findings add to the limited understanding of the association between dietary fatty acids and blood lipids in adolescents, taking into account the often-neglected context of the nutrients they replace. This was addressed by means of substitution models, which despite being theoretical, allow for a more integrative and disentangled interpretation of the results than single-nutrient models. Generally, in population-based samples, increasing dietary fat tends to be accompanied by a simultaneous reduction in CHO [19], and hence the associations here presented were calculated in the context of reducing CHO. Nevertheless, we are aware of the limitations of epidemiological studies in their ability to capture true dietary intake due to their reliance on subjective reports. The FFQ used in the present study was designed for the assessment of dietary intake over the past year in school-aged children, with special focus on fatty acid and antioxidant intakes. The estimated intakes underwent extensive quality control, and despite the possibility of reporting bias, the resulting data presented plausible values of total energy and macronutrient intakes. The applied FFQ has been used in previous studies and has successfully detected significant associations [22,34,43,65,66], which would likely not be the case if the method were flawed.

Another strength of the present study was the use of accelerometers, which allowed for an objective assessment of habitual daily PA. This is a rarely-available method in large epidemiological studies, and permitted a minute-by-minute classification of daily activity into different intensity levels, as well as the definition of tertiles based on the amount of time spent in each PA level. The stratification of our analyses into these subgroups adds novel information to the poorly understood interaction between dietary fatty acids and physical activity (including sedentary, lifestyle PA and MVPA) in lipoprotein metabolism.

It must be kept in mind that our findings are based on cross-sectional analyses, and hence the observed associations do not necessarily infer causality. Moreover, the assessment of diet and PA were not performed at exactly the same time, and hence analyses were not adjusted for season. Nevertheless, we observed no association between the season of dietary assessment and fatty acid intakes in bivariate analyses. An association was however observed between the season of PA assessment and lifestyle PA, with greater PA levels occurring in spring and summer compared to winter. We therefore cannot exclude the possibility of residual confounding by seasonal differences related to PA or lack thereof, such as sun exposure and differing levels of vitamin D, which have been suggested to play a beneficial role in glycemic control [67]. Nonetheless, sensitivity analyses with further adjustment for season did not alter the observed associations, except in males in the highest sedentary tertile, where the inverse association of MUFA with TAG no longer reached statistical significance (*p* = 0.032, data not shown). Finally, as often occurring in cohort studies, children of lower social classes were under-represented in our study population. Although we adjusted for parental education, our findings may not be representative of the study area. With this non-random loss-to-follow-up, adolescents in our study might present healthier blood lipid profiles as well as better lifestyle habits than the general population [34]. The fact that associations were nevertheless detected in our study population emphasizes the relevance of the present study findings and the need for further investigation.

## 5. Conclusions

Our findings suggest that replacing dietary CHO with MUFA (both sexes) or n-3 PUFA (only females) may induce beneficial changes in the blood lipid profile of adolescents. Adolescents, and particularly females, who are the least active in their daily lives, seem to be more susceptible to changes in diet involving the substitution of dietary fatty acids for CHO. Our results highlight the complexity of the body as a biological system, where the effects of diet on the lipid profile might vary in an activity-specific manner, emphasizing possible synergistic roles of diet and PA, especially lifestyle PA.

## Figures and Tables

**Table 1:** Study population characteristics.

	Males (*N* = 365)	Females (*N* = 472)	*p*-Value ^a^
Blood lipids			
LDL cholesterol (mmol/L)	2.22 (1.8; 2.7)	2.35 (2.0; 2.8)	**0.008**
HDL cholesterol (mmol/L)	1.40 (0.36)	1.57 (0.36)	**<0.001** ^b^
TAG (mmol/L)	1.04 (0.7; 1.4)	0.98 (0.7; 1.3)	0.139
Total:HDL	3.00 (2.6; 3.6)	2.87 (2.5; 3.3)	**0.002**
Fatty acids			
SFA (% EI)	13.0 (2.9)	12.7 (3.1)	0.290 ^b^
MUFA (% EI)	11.2 (2.5)	10.9 (2.7)	0.100 ^b^
n-6 PUFA (% EI)	3.9 (3.3; 4.7)	3.9 (3.3; 4.8)	0.712
n-3 PUFA (% EI)	0.6 (0.5; 0.7)	0.6 (0.5; 0.7)	0.425
Physical activity			
Sedentary (min/d)	583 (530; 632)	599 (563; 644)	**<0.001**
Lifestyle PA (min/d)	258 (222; 296)	241 (209; 275)	**<0.001**
MVPA (min/d)	43 (31; 61)	35 (25; 47)	**<0.001**
Covariates			
Age (years)	15.2 (0.3)	15.2 (0.3)	0.773 ^b^
BMI (kg/m^2^)	19.9 (18.5; 22.1)	20.3 (18.7; 22.1)	0.135
Parental education (high)	272 (74.5)	345 (73.1)	0.699 ^c^
Fasting blood (yes)	187 (51.2)	203 (43.0)	**0.022** ^c^
Daily calories (kcal)	2397 (668)	1848 (563)	**<0.001** ^b^
Total carbohydrate (% EI)	52.6 (6.7)	53.3 (7.5)	0.127 ^b^
Total fat (% EI)	31.3 (5.7)	30.9 (6.4)	0.350 ^b^
Total protein (% EI)	15 (2.5)	14.6 (2.9)	**0.047** ^b^
Study			0.089 ^c^
GINI intervention	117 (32.1)	174 (36.9)	
GINI observation	135 (37.0)	183 (38.8)	
LISA	113 (31.0)	115 (24.4)	
Region			**0.001** ^c^
Munich	260 (71.2)	283 (60.0)	
Wesel	105 (28.8)	189 (40.0)	
Pubertal stage			**<0.001** ^c^
Early	21 (5.8)	0 (0.0)	
Mid	134 (36.7)	20 (4.2)	
Late	207 (56.7)	377 (79.9)	
Post	3 (0.8)	75 (15.9)	

Presented values are means (standard deviation), medians (25th percentile; 75th percentile) or counts (%); LDL: Low-densitiy lipoprotein; HDL: High-density lipoprotein; TAG: Triglycerides; Total:HDL: Total cholesterol to HDL ratio; SFA: Saturated fatty acids; MUFA: Monounsaturated fatty acids; PUFA: Polyunsaturated fatty acids; PA: Physical activity; MVPA: Moderate-to-vigorous physical activity; Parental education, high: >10th grade; Fasting blood, yes: Blood collected was in the fasted state; ^a^ tested by Wilcoxon rank sum test; ^b^ tested by Student’s *t*-test; ^c^ tested by χ^2^-test; Significant differences between females and males are marked in bold (*p*-value < 0.05).

**Table 2:** Association of different types of fatty acids (when theoretically replacing dietary carbohydrates (CHO)) with blood lipid parameters.

	SFA			MUFA			n-6 PUFA			n-3 PUFA		
**Females**												
LDL	MR	95% CI	*p*-value	MR	95% CI	*p*-value	MR	95% CI	*p*-value	MR	95% CI	*p*-value
	1.00	(0.97, 1.04)	0.814	1.00	(0.96, 1.04)	0.902	0.97	(0.93, 1.01)	0.184	1.02	(0.98, 1.06)	0.304
HDL	β	95% CI	*p*-value	β	95% CI	*p*-value	β	95% CI	*p*-value	β	95% CI	*p*-value
	0.01	(−0.04, 0.05)	0.751	0.02	(−0.03, 0.06)	0.543	−0.04	(−0.09, 0.01)	0.120	0.07	(0.03; 0.12)	**0.002**
TAG	MR	95% CI	*p*-value	MR	95% CI	*p*-value	MR	95% CI	*p*-value	MR	95% CI	*p*-value
	0.98	(0.93, 1.04)	0.544	0.94	(0.89, 1.00)	**0.049**	1.05	(0.99, 1.12)	0.089	0.98	(0.93, 1.03)	0.419
Total:HDL	MR	95% CI	*p*-value	MR	95% CI	*p*-value	MR	95% CI	*p*-value	MR	95% CI	*p*-value
	1.00	(0.97, 1.02)	0.760	0.99	(0.96, 1.02)	0.449	1.00	(0.97, 1.03)	0.902	0.980	(0.95, 1.01)	0.163
**Males**												
LDL	MR	95% CI	*p*-value	MR	95% CI	*p*-value	MR	95% CI	*p*-value	MR	95% CI	*p*-value
	1.01	(0.96, 1.07)	0.603	1.00	(0.95, 1.05)	0.902	1.00	(0.94, 1.05)	0.932	1.00	(0.95, 1.05)	0.915
HDL	β	95% CI	*p*-value	β	95% CI	*p*-value	β	95% CI	*p*-value	β	95% CI	*p*-value
	0.05	(−0.01, 0.11)	0.078	0.05	(−0.00, 0.11)	0.064	0.00	(−0.06, 0.06)	0.972	−0.02	(−0.08; 0.03)	0.384
TAG	MR	95% CI	*p*-value	MR	95% CI	*p*-value	MR	95% CI	*p*-value	MR	95% CI	*p*-value
	0.95	(0.88, 1.01)	0.113	0.87	(0.82, 0.94)	**<0.001**	1.04	(0.97, 1.12)	0.268	1.03	(0.96, 1.09)	0.401
Total:HDL	MR	95% CI	*p*-value	MR	95% CI	*p*-value	MR	95% CI	*p*-value	MR	95% CI	*p*-value
	0.99	(0.94, 1.03)	0.542	0.98	(0.93, 1.02)	0.326	0.99	(0.94, 1.04)	0.618	1.02	(0.98, 1.07)	0.381

MR: Means ratio; β: Beta coefficient; LDL: Low-density lipoprotein cholesterol; HDL: High-density lipoprotein cholesterol; TAG: Triglycerides; Total:HDL: Total cholesterol to HDL ratio; SFA: Saturated fatty acids; MUFA: Monounsaturated fatty acids; PUFA: Polyunsaturated fatty acids; significant associations marked in bold (*p*-value < 0.05).

## Supplementary Materials

The following are available online at http://www.mdpi.com/2072-6643/10/10/1372/s1: Table S1: Association of different types of fatty acids (when theoretically replacing dietary CHO) with blood lipid parameters, additionally adjusting for different levels of physical activity; Table S2: Association of different types of fatty acids (when theoretically replacing dietary CHO) with blood lipid parameters in females, stratified by sedentary, lifestyle PA and MVPA tertiles; Table S3: Association of different types of fatty acids (when theoretically replacing dietary CHO) with blood lipid parameters in males, stratified by sedentary, lifestyle PA and MVPA tertiles.
