# Supplementary material for: Association of Dietary Fatty Acids with Blood Lipids is Modified by Physical Activity in Adolescents: Results from the GINIplus and LISA Birth Cohort Studies
Source: Nutrients. 2018 Sep 25;10(10):1372. doi: 10.3390/nu10101372 (PMC6213167; doi:10.3390/nu10101372)
Supplement: Supplementary file 1 [file nutrients-10-01372-s001.zip › nutrients-355967-supplementary/Supplementary Table S1.doc]

**Supplementary Table S1.** Association of different types of fatty acids (when theoretically replacing dietary CHO) with blood lipid parameters, additionally adjusting for different levels of physical activity.

|  | **SFA** | | | **MUFA** | | | **n-6 PUFA** | | | **n-3 PUFA** | | |
| --- | --- | --- | --- | --- | --- | --- | --- | --- | --- | --- | --- | --- |
| **Females** |  |  |  |  |  |  |  |  |  |  |  |  |
|  | MR | 95%CI | *p*-Value | MR | 95%CI | *p*-Value | MR | 95%CI | *p*-Value | MR | 95%CI | *p*-Value |
| LDL | 1.00 | (0.97, 1.04) | 0.814 | 1.00 | (0.96, 1.04) | 0.902 | 0.97 | (0.93, 1.01) | 0.184 | 1.02 | (0.98, 1.06) | 0.304 |
| +Sedentary | 1.00 | (0.97, 1.04) | 0.803 | 1.00 | (0.96, 1.04) | 0.921 | 0.97 | (0.93, 1.01) | 0.187 | 1.02 | (0.98, 1.06) | 0.322 |
| +Light PA | 1.00 | (0.97, 1.04) | 0.809 | 1.00 | (0.96, 1.04) | 0.898 | 0.97 | (0.93, 1.01) | 0.174 | 1.02 | (0.98, 1.06) | 0.285 |
| +MVPA | 1.00 | (0.97, 1.04) | 0.854 | 1.00 | (0.96, 1.04) | 0.918 | 0.97 | (0.93, 1.01) | 0.193 | 1.02 | (0.98, 1.06) | 0.281 |
|  | β | 95%CI | *p*-Value | β | 95%CI | *p*-Value | β | 95%CI | *p*-Value | Β | 95%CI | *p*-Value |
| HDL | 0.01 | (–0.04; 0.05) | 0.751 | 0.02 | (–0.03; 0.06) | 0.543 | –0.04 | (–0.09; 0.01) | 0.120 | 0.07 | (0.03; 0.12) | **0.002** |
| +Sedentary | 0.01 | (–0.04; 0.05) | 0.742 | 0.01 | (–0.03; 0.06) | 0.557 | –0.04 | (–0.09; 0.01) | 0.122 | 0.07 | (0.03; 0.12) | **0.002** |
| +Light PA | 0.01 | (–0.04; 0.05) | 0.745 | 0.02 | (–0.03; 0.06) | 0.539 | –0.04 | (–0.09; 0.01) | 0.111 | 0.07 | (0.03; 0.12) | **0.002** |
| +MVPA | 0.01 | (–0.04; 0.05) | 0.710 | 0.02 | (–0.03; 0.06) | 0.530 | –0.04 | (–0.09; 0.01) | 0.112 | 0.07 | (0.03; 0.12) | **0.002** |
|  | MR | 95%CI | *p*-Value | MR | 95%CI | *p*-Value | MR | 95%CI | *p*-Value | MR | 95%CI | *p*-Value |
| TAG | 0.98 | (0.93, 1.04) | 0.544 | 0.94 | (0.89, 1.00) | **0.049** | 1.05 | (0.99, 1.12) | 0.089 | 0.98 | (0.93, 1.03) | 0.419 |
| +Sedentary | 0.98 | (0.93, 1.04) | 0.544 | 0.94 | (0.89, 1.00) | **0.049** | 1.05 | (0.99, 1.12) | 0.089 | 0.98 | (0.93, 1.03) | 0.422 |
| +Light PA | 0.98 | (0.93, 1.04) | 0.541 | 0.94 | (0.89, 1.00) | **0.048** | 1.05 | (0.99, 1.12) | 0.084 | 0.98 | (0.93, 1.03) | 0.400 |
| +MVPA | 0.98 | (0.93, 1.04) | 0.541 | 0.94 | (0.89, 1.00) | **0.049** | 1.05 | (0.99, 1.12) | 0.088 | 0.98 | (0.93, 1.03) | 0.424 |
|  | MR | 95%CI | *p*-Value | MR | 95%CI | *p*-Value | MR | 95%CI | *p*-Value | MR | 95%CI | *p*-Value |
| Total:HDL | 1.00 | (0.97, 1.02) | 0.760 | 0.99 | (0.96, 1.02) | 0.449 | 1.00 | (0.97, 1.03) | 0.902 | 0.98 | (0.95, 1.01) | 0.163 |
| +Sedentary | 1.00 | (0.97, 1.02) | 0.762 | 0.99 | (0.96, 1.02) | 0.448 | 1.00 | (0.97, 1.03) | 0.904 | 0.98 | (0.95, 1.01) | 0.163 |
| +Light PA | 1.00 | (0.97, 1.02) | 0.760 | 0.99 | (0.96, 1.02) | 0.449 | 1.00 | (0.97, 1.03) | 0.904 | 0.98 | (0.95, 1.01) | 0.164 |
| +MVPA | 0.99 | (0.97, 1.02) | 0.704 | 0.99 | (0.96, 1.02) | 0.432 | 1.00 | (0.97, 1.03) | 0.935 | 0.98 | (0.95, 1.01) | 0.186 |
| **Males** |  |  |  |  |  |  |  |  |  |  |  |  |
|  | MR | 95%CI | *p*-Value | MR | 95%CI | *p*-Value | MR | 95%CI | *p*-Value | MR | 95%CI | *p*-Value |
| LDL | 1.01 | (0.96, 1.07) | 0.603 | 1.00 | (0.95, 1.05) | 0.902 | 1.00 | (0.94, 1.05) | 0.932 | 1.00 | (0.95, 1.05) | 0.915 |
| +Sedentary | 1.01 | (0.96, 1.07) | 0.579 | 1.00 | (0.95, 1.05) | 0.915 | 1.00 | (0.94, 1.05) | 0.935 | 1.00 | (0.95, 1.04) | 0.884 |
| +Light PA | 1.01 | (0.96, 1.07) | 0.584 | 1.00 | (0.95, 1.05) | 0.915 | 1.00 | (0.94, 1.05) | 0.939 | 1.00 | (0.95, 1.04) | 0.900 |
| +MVPA | 1.01 | (0.96, 1.07) | 0.589 | 1.00 | (0.95, 1.06) | 0.885 | 1.00 | (0.94, 1.05) | 0.928 | 1.00 | (0.95, 1.05) | 0.908 |
|  | β | 95%CI | *p*-Value | β | 95%CI | *p*-Value | β | 95%CI | *p*-Value | β | 95%CI | *p*-Value |
| HDL | 0.05 | (–0.01; 0.11) | 0.078 | 0.05 | (–0.00; 0.11) | 0.064 | 0.00 | (–0.06; 0.06) | 0.972 | –0.02 | (–0.08; 0.03) | 0.384 |
| +Sedentary | 0.05 | (–0.01; 0.11) | 0.084 | 0.05 | (–0.00; 0.11) | 0.063 | 0.00 | (–0.06; 0.06) | 0.975 | –0.02 | (–0.07; 0.03) | 0.401 |
| +Light PA | 0.05 | (–0.01; 0.11) | 0.078 | 0.05 | (–0.00; 0.11) | 0.065 | 0.00 | (–0.06; 0.06) | 0.971 | –0.02 | (–0.08; 0.03) | 0.383 |
| +MVPA | 0.06 | (–0.00; 0.11) | 0.057 | 0.06 | (0.00; 0.11) | **0.047** | 0.00 | (–0.06; 0.06) | 0.997 | –0.02 | (–0.08; 0.03) | 0.355 |
|  | MR | 95%CI | *p*-Value | MR | 95%CI | *p*-Value | MR | 95%CI | *p*-Value | MR | 95%CI | *p*-Value |
| TAG | 0.95 | (0.88, 1.01) | 0.113 | 0.87 | (0.82, 0.94) | **<0.001** | 1.04 | (0.97, 1.12) | 0.268 | 1.03 | (0.96, 1.09) | 0.401 |
| +Sedentary | 0.94 | (0.88, 1.01) | 0.096 | 0.88 | (0.82, 0.94) | **<0.001** | 1.04 | (0.97, 1.12) | 0.271 | 1.03 | (0.97, 1.10) | 0.354 |
| +Light PA | 0.94 | (0.88, 1.01) | 0.103 | 0.88 | (0.82, 0.94) | **<0.001** | 1.04 | (0.97, 1.12) | 0.274 | 1.03 | (0.97, 1.10) | 0.385 |
| +MVPA | 0.94 | (0.88, 1.01) | 0.085 | 0.87 | (0.81, 0.93) | **<0.001** | 1.04 | (0.97, 1.13) | 0.254 | 1.03 | (0.97, 1.10) | 0.373 |
|  | MR | 95%CI | *p*-Value | MR | 95%CI | *p*-Value | MR | 95%CI | *p*-Value | MR | 95%CI | *p*-Value |
| Total:HDL | 0.99 | (0.94, 1.03) | 0.542 | 0.98 | (0.93, 1.02) | 0.326 | 0.99 | (0.94, 1.04) | 0.618 | 1.02 | (0.98, 1.07) | 0.381 |
| +Sedentary | 0.99 | (0.94, 1.03) | 0.580 | 0.98 | (0.93, 1.02) | 0.315 | 0.99 | (0.94, 1.04) | 0.622 | 1.02 | (0.97, 1.06) | 0.415 |
| +Light PA | 0.99 | (0.94, 1.03) | 0.576 | 0.98 | (0.93, 1.02) | 0.313 | 0.99 | (0.94, 1.04) | 0.628 | 1.02 | (0.98, 1.06) | 0.398 |
| +MVPA | 0.98 | (0.94, 1.03) | 0.439 | 0.97 | (0.93, 1.02) | 0.252 | 0.99 | (0.94, 1.04) | 0.642 | 1.02 | (0.98, 1.07) | 0.348 |

MR: Means ratio; β: Beta coefficient; LDL: Low-density lipoprotein; HDL: High-density lipoprotein; TAG: Triglycerides; Total:HDL: Total cholesterol to HDL ratio; SFA: Saturated fatty acids; MUFA: Monounsaturated fatty acids; PUFA: Polyunsaturated fatty acids; PA: Physical activity; MVP: Moderate-to-vigorous physical activity; significant associations in bold (*p*-value < 0.05).
