# Supplementary material for: Association of Dietary Fatty Acids with Blood Lipids is Modified by Physical Activity in Adolescents: Results from the GINIplus and LISA Birth Cohort Studies
Source: Nutrients. 2018 Sep 25;10(10):1372. doi: 10.3390/nu10101372 (PMC6213167; doi:10.3390/nu10101372)
Supplement: Supplementary file 1 [file nutrients-10-01372-s001.zip › nutrients-355967-supplementary/Supplementary Table S2.doc]

**Supplementary Table S2.** Association of different types of fatty acids (when theoretically replacing dietary CHO) with blood lipid parameters in females, stratified by sedentary, lifestyle PA and MVPA tertiles [minutes].

| **Sedentary** |  | **Tertile 1 [346.0–579.0]** | | | **Tertile 2 [579.7–626.9]** | | | **Tertile 3 [627.0–823.4]** | | |  |
| --- | --- | --- | --- | --- | --- | --- | --- | --- | --- | --- | --- |
| **Exposure** | **Outcome** | MR or β | 95% CI | *p*-Value | MR or β | 95% CI | *p*-Value | MR or β | 95% CI | *p*-Value | *p*-int |
| SFA | LDL | 0.99 | (0.92, 1.06) | 0.692 | 1.02 | (0.96, 1.09) | 0.508 | 0.98 | (0.92, 1.04) | 0.533 | 0.471 |
|  | HDL | –0.06 | (–0.15; 0.02) | 0.140 | 0.03 | (–0.05; 0.11) | 0.459 | 0.05 | (–0.04; 0.13) | 0.288 | **0.045** |
|  | TAG | 1.03 | (0.93, 1.15) | 0.533 | 0.97 | (0.89, 1.06) | 0.545 | 0.92 | (0.84, 1.01) | 0.089 | 0.606 |
|  | Total:HDL | 1.01 | (0.96, 1.07) | 0.598 | 0.99 | (0.94, 1.05) | 0.765 | 0.97 | (0.92, 1.02) | 0.291 | 0.576 |
| MUFA | LDL | 0.98 | (0.91, 1.06) | 0.612 | 1.01 | (0.95, 1.08) | 0.683 | 1.03 | (0.96, 1.11) | 0.445 | 0.422 |
|  | HDL | –0.02 | (–0.12; 0.07) | 0.589 | –0.01 | (–0.09; 0.07) | 0.842 | 0.08 | (–0.01; 0.18) | 0.086 | 0.160 |
|  | TAG | 0.99 | (0.88, 1.11) | 0.819 | 0.95 | (0.87, 1.04) | 0.268 | 0.91 | (0.82, 1.02) | 0.097 | 0.793 |
|  | Total:HDL | 0.99 | (0.94, 1.05) | 0.764 | 1.00 | (0.95, 1.06) | 0.926 | 0.98 | (0.93, 1.04) | 0.596 | 0.978 |
| n-6 PUFA | LDL | 0.98 | (0.90, 1.06) | 0.632 | 0.97 | (0.90, 1.04) | 0.381 | 0.93 | (0.86, 1.00) | 0.045 | 0.706 |
|  | HDL | –0.08 | (–0.18; 0.01) | 0.085 | –0.01 | (–0.1; 0.08) | 0.818 | –0.07 | (–0.16; 0.03) | 0.167 | 0.247 |
|  | TAG | 1.05 | (0.93, 1.18) | 0.462 | 1.04 | (0.94, 1.15) | 0.437 | 1.07 | (0.96, 1.18) | 0.232 | 0.413 |
|  | Total:HDL | 1.02 | (0.96, 1.09) | 0.435 | 0.98 | (0.92, 1.04) | 0.482 | 0.98 | (0.92, 1.04) | 0.520 | 0.582 |
| n-3 PUFA | LDL | 0.99 | (0.91, 1.06) | 0.710 | 1.01 | (0.96, 1.06) | 0.735 | 1.08 | (1.01, 1.15) | 0.026 | 0.094 |
|  | HDL | 0.10 | (0.01; 0.19) | 0.032 | 0.01 | (–0.06; 0.07) | 0.860 | 0.12 | (0.03; 0.20) | **0.007** | 0.459 |
|  | TAG | 0.96 | (0.85, 1.08) | 0.473 | 0.97 | (0.91, 1.05) | 0.469 | 1.01 | (0.92, 1.11) | 0.854 | 0.282 |
|  | Total:HDL | 0.95 | (0.89, 1.00) | 0.071 | 1.00 | (0.96, 1.05) | 0.877 | 0.99 | (0.94, 1.04) | 0.682 | 0.487 |
| **Lifestyle PA** |  | **Tertile 1 [102.4–219.8]** | | | **Tertile 2 [220.6–264.5]** | | | **Tertile 3 [265.9–435.3]** | | |  |
| **Exposure** | **Outcome** | MR or β | 95% CI | *p*-Value | MR or β | 95% CI | *p*-Value | MR or β | 95% CI | *p*-Value | *p*-int |
| SFA | LDL | 0.98 | (0.92, 1.03) | 0.409 | 1.00 | (0.93, 1.07) | 0.959 | 1.02 | (0.95, 1.09) | 0.647 | 0.574 |
|  | HDL | 0.01 | (–0.07; 0.08) | 0.863 | 0.01 | (–0.07; 0.09) | 0.807 | 0.02 | (–0.07; 0.12) | 0.692 | 0.928 |
|  | TAG | 0.91 | (0.85, 0.98) | **0.013** | 0.97 | (0.89, 1.07) | 0.572 | 1.03 | (0.92, 1.14) | 0.627 | 0.434 |
|  | Total:HDL | 0.98 | (0.94, 1.03) | 0.396 | 0.99 | (0.94, 1.05) | 0.793 | 0.99 | (0.94, 1.05) | 0.836 | 0.777 |
| MUFA | LDL | 0.99 | (0.93, 1.06) | 0.880 | 0.99 | (0.92, 1.07) | 0.763 | 1.00 | (0.94, 1.07) | 0.956 | 0.425 |
|  | HDL | 0.05 | (–0.03; 0.13) | 0.243 | 0.00 | (–0.09; 0.08) | 0.921 | 0.02 | (–0.06; 0.11) | 0.586 | 0.731 |
|  | TAG | 0.88 | (0.81, 0.96) | **0.004** | 0.92 | (0.83, 1.02) | 0.102 | 0.99 | (0.89, 1.09) | 0.797 | 0.499 |
|  | Total:HDL | 0.98 | (0.93, 1.03) | 0.393 | 0.99 | (0.93, 1.04) | 0.616 | 0.98 | (0.93, 1.04) | 0.464 | 0.983 |
| n-6 PUFA | LDL | 0.94 | (0.87, 1.01) | 0.075 | 0.95 | (0.88, 1.02) | 0.142 | 1.02 | (0.94, 1.10) | 0.665 | 0.989 |
|  | HDL | –0.11 | (–0.20; –0.02) | 0.022 | 0.05 | (–0.04; 0.14) | 0.256 | -0.05 | (–0.15; 0.05) | 0.329 | 0.989 |
|  | TAG | 1.11 | (1.01, 1.22) | 0.030 | 0.98 | (0.88, 1.08) | 0.626 | 1.03 | (0.92, 1.16) | 0.592 | 0.204 |
|  | Total:HDL | 1.01 | (0.95, 1.06) | 0.824 | 0.94 | (0.89, 1.00) | 0.037 | 1.02 | (0.96, 1.09) | 0.457 | 0.758 |
| n-3 PUFA | LDL | 1.10 | (1.04, 1.16) | **0.001** | 0.96 | (0.89, 1.04) | 0.279 | 0.98 | (0.92, 1.05) | 0.660 | 0.062 |
|  | HDL | 0.13 | (0.06; 0.20) | **<0.001** | –0.01 | (–0.10; 0.09) | 0.878 | 0.03 | (–0.06; 0.12) | 0.507 | 0.402 |
|  | TAG | 0.98 | (0.91, 1.06) | 0.635 | 1.09 | (0.98, 1.21) | 0.135 | 0.94 | (0.85, 1.04) | 0.229 | 0.127 |
|  | Total:HDL | 0.99 | (0.95, 1.04) | 0.684 | 0.99 | (0.93, 1.05) | 0.736 | 0.98 | (0.93, 1.04) | 0.489 | 0.416 |
| **MVPA** |  | **Tertile 1 [6.7–29.0]** | | | **Tertile 2 [29.1–42.9]** | | | **Tertile 3 [43.0–171.9]** | | |  |
| **Exposure** | **Outcome** | MR or β | 95% CI | *p*-Value | MR or β | 95% CI | *p*-Value | MR or β | 95% CI | *p*-Value | *p*-int |
| SFA | LDL | 1.00 | (0.94, 1.07) | 0.924 | 0.99 | (0.94, 1.06) | 0.864 | 0.99 | (0.91, 1.06) | 0.703 | 0.366 |
|  | HDL | –0.01 | (–0.08; 0.07) | 0.891 | –0.01 | (–0.10; 0.08) | 0.774 | 0.00 | (–0.08; 0.09) | 0.958 | 0.604 |
|  | TAG | 0.98 | (0.90, 1.07) | 0.662 | 0.99 | (0.90, 1.09) | 0.831 | 0.94 | (0.85, 1.04) | 0.228 | 0.511 |
|  | Total:HDL | 1.01 | (0.96, 1.06) | 0.784 | 1.00 | (0.94, 1.06) | 0.923 | 0.98 | (0.93, 1.03) | 0.358 | 0.676 |
| MUFA | LDL | 1.02 | (0.95, 1.09) | 0.569 | 0.99 | (0.93, 1.06) | 0.817 | 0.98 | (0.90, 1.07) | 0.616 | 0.317 |
|  | HDL | –0.01 | (–0.10; 0.07) | 0.774 | –0.02 | (–0.11; 0.08) | 0.711 | 0.02 | (–0.08; 0.11) | 0.733 | 0.218 |
|  | TAG | 0.93 | (0.85, 1.02) | 0.136 | 0.97 | (0.88, 1.08) | 0.623 | 0.90 | (0.80, 1.02) | 0.096 | 0.680 |
|  | Total:HDL | 1.02 | (0.97, 1.08) | 0.472 | 1.00 | (0.94, 1.06) | 0.997 | 0.96 | (0.90, 1.01) | 0.144 | 0.844 |
| n-6 PUFA | LDL | 0.94 | (0.88, 1.01) | 0.111 | 1.03 | (0.96, 1.10) | 0.453 | 0.98 | (0.90, 1.07) | 0.669 | 0.679 |
|  | HDL | –0.02 | (–0.10; 0.07) | 0.731 | 0.00 | (–0.11; 0.10) | 0.956 | –0.07 | (–0.16; 0.02) | 0.144 | 0.295 |
|  | TAG | 1.06 | (0.96, 1.16) | 0.256 | 1.04 | (0.93, 1.17) | 0.472 | 1.09 | (0.97, 1.22) | 0.143 | 0.824 |
|  | Total:HDL | 0.97 | (0.92, 1.03) | 0.286 | 1.01 | (0.94, 1.08) | 0.849 | 1.03 | (0.97, 1.09) | 0.354 | 0.896 |
| n-3 PUFA | LDL | 1.06 | (1.00, 1.13) | 0.066 | 1.01 | (0.96, 1.07) | 0.613 | 0.99 | (0.92, 1.07) | 0.795 | 0.622 |
|  | HDL | 0.08 | (0.00; 0.16) | 0.042 | 0.06 | (–0.01; 0.14) | 0.107 | 0.07 | (–0.01; 0.16) | 0.089 | 0.719 |
|  | TAG | 0.99 | (0.91, 1.08) | 0.863 | 0.99 | (0.91, 1.07) | 0.751 | 0.94 | (0.85, 1.05) | 0.268 | 0.714 |
|  | Total:HDL | 0.99 | (0.94, 1.05) | 0.826 | 0.98 | (0.94, 1.03) | 0.530 | 0.95 | (0.90, 1.00) | 0.074 | 0.579 |

MR: Means ratio; β: Beta coefficient; LDL: Low-density lipoprotein; HDL: High-density lipoprotein; TAG: Triglycerides; Total:HDL: Total cholesterol to HDL ratio; SFA: Saturated fatty acids; MUFA: Monounsaturated fatty acids; PUFA: Polyunsaturated fatty acids; *p*-int: *p*-value for interaction. Significant associations in bold (*p*-Value < 0.017; *p*-int < 0.05).
