# Supplementary material for: Association of Dietary Fatty Acids with Blood Lipids is Modified by Physical Activity in Adolescents: Results from the GINIplus and LISA Birth Cohort Studies
Source: Nutrients. 2018 Sep 25;10(10):1372. doi: 10.3390/nu10101372 (PMC6213167; doi:10.3390/nu10101372)
Supplement: Supplementary file 1 [file nutrients-10-01372-s001.zip › nutrients-355967-supplementary/Supplementary Table S3.doc]

**Supplementary Table S3.** Association of different types of fatty acids (when theoretically replacing dietary CHO) with blood lipid parameters in males, stratified by sedentary, lifestyle PA and MVPA tertiles [minutes].

| **Sedentary** |  | **Tertile 1 [373.1–546.2]** | | | **Tertile 2 [546.5–612.4]** | | | **Tertile 3 [612.4–810.9]** | | |  |
| --- | --- | --- | --- | --- | --- | --- | --- | --- | --- | --- | --- |
| **Exposure** | **Outcome** | MR or β | 95% CI | *p*-Value | MR or β | 95% CI | *p*-Value | MR or β | 95% CI | *p*-Value | *p*-int |
| SFA | LDL | 0.99 | (0.90, 1.09) | 0.824 | 1.03 | (0.93, 1.14) | 0.586 | 1.02 | (0.94, 1.11) | 0.588 | 0.637 |
|  | HDL | –0.02 | (–0.13; 0.10) | 0.795 | 0.06 | (–0.04; 0.17) | 0.246 | 0.08 | (–0.01; 0.18) | 0.067 | 0.495 |
|  | TAG | 0.98 | (0.87, 1.11) | 0.801 | 0.90 | (0.78, 1.04) | 0.155 | 0.95 | (0.84, 1.06) | 0.355 | 0.745 |
|  | Total:HDL | 1.02 | (0.91, 1.14) | 0.733 | 0.98 | (0.91, 1.07) | 0.703 | 0.96 | (0.90, 1.02) | 0.218 | 0.541 |
| MUFA | LDL | 0.98 | (0.91, 1.06) | 0.609 | 1.08 | (0.98, 1.20) | 0.115 | 0.97 | (0.88, 1.06) | 0.491 | 0.277 |
|  | HDL | 0.02 | (–0.08; 0.12) | 0.714 | 0.07 | (–0.03; 0.17) | 0.176 | 0.04 | (–0.06; 0.14) | 0.454 | 0.526 |
|  | TAG | 0.96 | (0.86, 1.06) | 0.382 | 0.85 | (0.74, 0.98) | 0.027 | 0.85 | (0.75, 0.97) | **0.015** | **0.045** |
|  | Total:HDL | 0.98 | (0.90, 1.08) | 0.741 | 1.01 | (0.93, 1.09) | 0.833 | 0.96 | (0.89, 1.03) | 0.258 | 0.066 |
| n-6 PUFA | LDL | 1.08 | (0.96, 1.22) | 0.211 | 0.99 | (0.92, 1.06) | 0.784 | 0.97 | (0.85, 1.10) | 0.600 | 0.383 |
|  | HDL | 0.00 | (–0.16; 0.16) | 0.986 | –0.04 | (–0.12; 0.03) | 0.242 | 0.07 | (–0.06; 0.21) | 0.270 | 0.760 |
|  | TAG | 1.01 | (0.86, 1.19) | 0.859 | 1.06 | (0.96, 1.17) | 0.227 | 1.06 | (0.89, 1.25) | 0.537 | 0.333 |
|  | Total:HDL | 1.06 | (0.91, 1.22) | 0.456 | 1.01 | (0.95, 1.07) | 0.787 | 0.92 | (0.84, 1.02) | 0.113 | 0.217 |
| n-3 PUFA | LDL | 0.93 | (0.85, 1.01) | 0.088 | 0.96 | (0.85, 1.07) | 0.456 | 1.03 | (0.96, 1.10) | 0.369 | 0.455 |
|  | HDL | 0.03 | (–0.08; 0.14) | 0.555 | –0.08 | (–0.20; 0.04) | 0.201 | –0.05 | (–0.12; 0.02) | 0.160 | 0.290 |
|  | TAG | 1.03 | (0.92, 1.15) | 0.646 | 1.06 | (0.91, 1.25) | 0.458 | 0.99 | (0.90, 1.08) | 0.815 | 0.724 |
|  | Total:HDL | 0.95 | (0.86, 1.05) | 0.345 | 1.02 | (0.93, 1.12) | 0.643 | 1.05 | (1.00, 1.11) | 0.052 | 0.487 |
| **Lifestyle PA** |  | **Tertile 1 [141.8–235.7]** | | | **Tertile 2 [235.9–280.7]** | | | **Tertile 3 [282.6–458.7]** | | |  |
| **Exposure** | **Outcome** | MR or β | 95% CI | *p*-Value | MR or β | 95% CI | *p*-Value | MR or β | 95% CI | *p*-Value | *p*-int |
| SFA | LDL | 1.02 | (0.94, 1.10) | 0.654 | 1.07 | (0.98, 1.17) | 0.147 | 0.95 | (0.86, 1.05) | 0.356 | 0.110 |
|  | HDL | 0.07 | (–0.01; 0.16) | 0.081 | 0.07 | (–0.03; 0.17) | 0.182 | –0.02 | (–0.14; 0.10) | 0.726 | 0.561 |
|  | TAG | 0.93 | (0.83, 1.03) | 0.155 | 1.00 | (0.89, 1.14) | 0.944 | 0.96 | (0.84, 1.09) | 0.495 | 0.823 |
|  | Total:HDL | 0.97 | (0.91, 1.03) | 0.272 | 1.02 | (0.95, 1.09) | 0.623 | 0.99 | (0.88, 1.10) | 0.792 | 0.656 |
| MUFA | LDL | 0.95 | (0.86, 1.04) | 0.284 | 1.07 | (0.98, 1.17) | 0.120 | 0.98 | (0.90, 1.06) | 0.570 | 0.822 |
|  | HDL | 0.07 | (–0.03; 0.17) | 0.156 | 0.08 | (–0.01; 0.18) | 0.092 | –0.01 | (–0.11; 0.09) | 0.884 | 0,297 |
|  | TAG | 0.86 | (0.75, 0.97) | **0.015** | 0.89 | (0.79, 1.01) | 0.069 | 0.93 | (0.84, 1.03) | 0.163 | 0.062 |
|  | Total:HDL | 0.93 | (0.87, 1.00) | 0.054 | 1.00 | (0.93, 1.07) | 0.966 | 0.98 | (0.90, 1.08) | 0.750 | 0.158 |
| n-6 PUFA | LDL | 1.05 | (0.95, 1.15) | 0.371 | 0.95 | (0.87, 1.05) | 0.337 | 0.98 | (0.88, 1.09) | 0.710 | 0.793 |
|  | HDL | 0.01 | (–0.09; 0.11) | 0.810 | –0.01 | (–0.12; 0.09) | 0.800 | –0.02 | (–0.16; 0.11) | 0.720 | 0.588 |
|  | TAG | 1.03 | (0.91, 1.17) | 0.644 | 1.04 | (0.91, 1.18) | 0.573 | 1.06 | (0.92, 1.21) | 0.423 | 0.308 |
|  | Total:HDL | 1.00 | (0.92, 1.07) | 0.929 | 0.98 | (0.91, 1.05) | 0.568 | 1.01 | (0.89, 1.14) | 0.871 | 0.476 |
| n-3 PUFA | LDL | 0.98 | (0.91, 1.05) | 0.496 | 1.02 | (0.94, 1.12) | 0.632 | 0.98 | (0.88, 1.09) | 0.729 | 0.378 |
|  | HDL | –0.05 | (–0.12; 0.02) | 0.144 | 0.01 | (–0.09; 0.11) | 0.849 | 0.01 | (–0.12; 0.13) | 0.940 | 0.307 |
|  | TAG | 1.03 | (0.94, 1.13) | 0.577 | 0.95 | (0.84, 1.07) | 0.370 | 1.06 | (0.93, 1.21) | 0.401 | 0.630 |
|  | Total:HDL | 1.03 | (0.97, 1.08) | 0.357 | 1.00 | (0.93, 1.07) | 0.936 | 1.00 | (0.89, 1.13) | 0.993 | 0.407 |
| **MVPA** |  | **Tertile 1 [5.2–35.9]** | | | **Tertile 2 [36.2–54.1]** | | | **Tertile 3 [54.4–133.8]** | | |  |
| **Exposure** | **Outcome** | MR or β | 95% CI | *p*-Value | MR or β | 95% CI | *p*-Value | MR or β | 95% CI | *p*-Value | *p*-int |
| SFA | LDL | 1.00 | (0.91, 1.10) | 0.988 | 1.05 | (0.95, 1.17) | 0.304 | 1.03 | (0.94, 1.11) | 0.548 | 0.475 |
|  | HDL | 0.07 | (–0.03; 0.17) | 0.184 | 0.06 | (–0.05; 0.17) | 0.260 | 0.01 | (–0.09; 0.11) | 0.830 | 0.559 |
|  | TAG | 0.97 | (0.86, 1.10) | 0.643 | 0.91 | (0.80, 1.03) | 0.152 | 0.98 | (0.87, 1.11) | 0.748 | 0.675 |
|  | Total:HDL | 0.96 | (0.89, 1.03) | 0.280 | 1.00 | (0.89, 1.12) | 0.963 | 1.01 | (0.94, 1.08) | 0.813 | 0.442 |
| MUFA | LDL | 1.02 | (0.91, 1.14) | 0.752 | 1.02 | (0.92, 1.12) | 0.757 | 1.00 | (0.94, 1.08) | 0.902 | 0.666 |
|  | HDL | 0.13 | (0.02; 0.24) | 0.027 | 0.04 | (–0.07; 0.14) | 0.464 | 0.00 | (–0.09; 0.08) | 0.943 | 0.627 |
|  | TAG | 0.88 | (0.77, 1.02) | 0.094 | 0.83 | (0.74, 0.94) | **0.003** | 0.95 | (0.86, 1.06) | 0.371 | 0.475 |
|  | Total:HDL | 0.93 | (0.86, 1.02) | 0.117 | 1.00 | (0.89, 1.11) | 0.952 | 1.01 | (0.95, 1.07) | 0.833 | 0.409 |
| n-6 PUFA | LDL | 0.98 | (0.90, 1.07) | 0.621 | 0.98 | (0.88, 1.09) | 0.704 | 1.02 | (0.92, 1.13) | 0.682 | 0.957 |
|  | HDL | –0.07 | (–0.16; 0.02) | 0.128 | 0.05 | (–0.07; 0.17) | 0.406 | 0.04 | (–0.08; 0.17) | 0.510 | 0.680 |
|  | TAG | 1.02 | (0.91, 1.14) | 0.740 | 1.08 | (0.94, 1.24) | 0.292 | 0.98 | (0.84, 1.14) | 0.808 | 0.396 |
|  | Total:HDL | 1.02 | (0.95, 1.09) | 0.600 | 0.92 | (0.81, 1.04) | 0.186 | 0.98 | (0.90, 1.07) | 0.658 | 0.943 |
| n-3 PUFA | LDL | 0.99 | (0.92, 1.07) | 0.867 | 1.03 | (0.94, 1.14) | 0.534 | 0.96 | (0.88, 1.05) | 0.347 | 0.575 |
|  | HDL | 0.01 | (–0.07; 0.09) | 0.842 | –0.07 | (–0.18; 0.03) | 0.166 | –0.05 | (–0.16; 0.06) | 0.379 | 0.480 |
|  | TAG | 0.94 | (0.85, 1.04) | 0.272 | 1.10 | (0.97, 1.24) | 0.138 | 1.11 | (0.97, 1.26) | 0.141 | 0.074 |
|  | Total:HDL | 1.00 | (0.95, 1.07) | 0.897 | 1.09 | (0.97, 1.21) | 0.143 | 1.02 | (0.94, 1.09) | 0.676 | 0.776 |

MR: Means ratio; β: Beta coefficient; LDL: Low-density lipoprotein; HDL: High-density lipoprotein; TAG: Triglycerides; Total:HDL: Total cholesterol to HDL ratio; SFA: Saturated fatty acids; MUFA: Monounsaturated fatty acids; PUFA: Polyunsaturated fatty acids; *p*-int: *p*-value for interaction. Significant associations in bold (*p*-Value < 0.017; *p*-int < 0.05).
